# High Incidence of Barotrauma in Patients With Severe Coronavirus Disease 2019

**DOI:** 10.1177/0885066621989959

**Published:** 2021-03-15

**Authors:** Michael R. Kahn, Richard L. Watson, Jay T. Thetford, Joseph Isaac Wong, Nader Kamangar

**Affiliations:** 1 Department of Medicine, 12222 UCLA-Olive View Medical Center, David Geffen School of Medicine at UCLA, Los Angeles, CA, USA; 2 Division of Pulmonary and Critical Care Medicine, Ronald Reagan 12222 UCLA Medical Center, David Geffen School of Medicine at UCLA, Los Angeles, CA, USA; 3 Division of Pulmonary and Critical Care Medicine, 12222 UCLA-Olive View Medical Center, David Geffen School of Medicine at UCLA, Los Angeles, CA, USA

**Keywords:** COVID-19, respiratory distress syndrome, adult, barotrauma, respiration, artificial, inflammation, physiology

## Abstract

**Objective.:**

To report the high incidence of barotrauma in critically ill patients admitted to the intensive care unit (ICU) with coronavirus disease 2019 (COVID-19) and to discuss its implications.

**Design.:**

Retrospective cohort study.

**Setting.:**

ICU of an academic county hospital in Los Angeles, CA admitted from March 15-June 20, 2020.

**Patients.:**

77 patients with COVID-19 pneumonia. 75 patients met inclusion criteria.

**Results.:**

21% of patients with severe COVID-19 sustained barotrauma (33% of patients receiving IMV, 8% of patients receiving (NIV). There were no differences between the barotrauma and non-barotrauma groups regarding demographics, illness severity, or medications received, nor tidal volume or average/peak airway pressures in those receiving IMV. In the barotrauma group there was a greater proportion of patients receiving therapeutic anticoagulation (81% vs. 47%, p = 0.023) and ventilated using airway pressure release ventilation mode (13% vs. 0%, p = 0.043). Barotrauma was associated with increased likelihood of receiving a tracheostomy (OR 2.58 [0.23-4.9], p = 0.018]), longer median ICU length of stay (17 days vs. 7 days, p = 0.03), and longer median length of hospitalization (26 days vs. 14 days, p < 0.001). There was also a trend toward prolonged median duration of IMV (12.5 days vs 7 days, p = 0.13) and higher average mortality (56% vs 37%, p = 0.25).

**Conclusions.:**

Barotrauma is seen in 5-12% of patients with ARDS receiving IMV and is exceedingly rare in patients receiving NIV. We report a high incidence of barotrauma observed in critically ill patients with COVID-19 requiring either NIV or IMV. While there was a trend toward increased mortality in patients with barotrauma, this did not reach statistical significance. The increased incidence of barotrauma with COVID-19 may be a product of the pathophysiology of this disease state and a heightened inflammatory response causing rampant acute lung injury. Evidence-based medicine and lung-protective ventilation should remain the mainstay of treatment.

## Introduction

The search for effective treatments for coronavirus disease 2019 (COVID-19) has become a priority in the medical community since the pandemic began. Meanwhile, invasive mechanical ventilation (IMV) continues to be a mainstay of treatment for severe respiratory failure. Despite novel therapies and lung-protective ventilation, mortality remains high for patients requiring IMV, often due to complications from sedation, nosocomial infections, and barotrauma.^1–3^ The high mortality associated with each of these risks makes understanding the sequelae of severe COVID-19 in the critical care setting all the more important.^4–6^

Barotrauma, defined as the presence of air outside the pleural surface of the lung, is a known complication of the acute respiratory distress syndrome (ARDS), occurring in 8-11% of all cases.^6–11^ Since the development of the ARDSnet protocol, low tidal volume ventilation at 6-8cc/kg based on ideal body weight (IBW) has reduced these rates to 5-8%.^8–11^ Notably, barotrauma is an exceedingly rare complication of patients who are receiving non-invasive ventilation (NIV) and is typically only seen in patients who are chronically using these therapies on the order of months to years.^7,12–14^ However, little data exists on the incidence of barotrauma in critically ill patients with COVID-19.

Initial retrospective data from China suggest that barotrauma was seen in as few as 3% of patients with COVID-19 receiving IMV (2% of patients total) whereas a subsequent study reported that up to 19% of patients with COVID-19 receiving IMV had evidence of extrapleural air on imaging, sometimes without clinical correlation.^15,16^ It is difficult to fully assess the disparity between these early reports; however, given that COVID-19 is still a relatively new disease process, and looking at data from other viral respiratory illnesses, the true incidence of barotrauma in COVID-19 is likely much greater than initially thought. For example, in severe acute respiratory syndrome-coronavirus 1 (SARS-CoV1) barotrauma was seen in 34% of patients receiving IMV and up to 15% of patients receiving NIV.^17–19^ Barotrauma was seen in 30% of patients receiving IMV diagnosed with Middle East Respiratory Syndrome coronavirus (MERS-CoV), 44% of patients treated for influenza A H7N9 (H7N9), and 8% of patients treated for influenza A H1N1 (H1N1).^20,21^

In this single center study, we examine a large sample of critically ill patients in order to better characterize the incidence of barotrauma in patients with COVID-19 respiratory failure, identify the risk factors associated with barotrauma, and determine its impact on patient mortality and length of hospital stay. In analyzing the data, this retrospective study also seeks to better illustrate the theoretical mechanisms of inflammatory processes in COVID-19 with clinical findings.

## Materials and Methods

### Study Design and Participants

This is a retrospective, observational cohort study conducted at a single center, the Olive View-UCLA Medical Center (OV-UCLA), an academic county hospital in Los Angeles (Sylmar), CA. A record of all patients admitted to the OV-UCLA adult intensive care unit (ICU) diagnosed with COVID-19, defined by a positive reverse-transcriptase-polymerase-chain-reaction (RT-PCR) for severe acute respiratory syndrome-coronavirus 2 (SARS-CoV2) by nasopharyngeal (NP) swab, was maintained in the electronic medical record starting 03/15/2020. All patients admitted to the hospital from this date forward received NP testing in the Emergency Department prior to being transported in the ICU in accordance with local infection control protocol.

We included all consecutive patients older than 18 years with confirmed COVID-19 who were admitted to the ICU between March 15 and June 15, 2020, with the following inclusion criteria in the first 24 h after admission: (1) mild to severe ARDS with partial pressure of arterial blood oxygen to fraction of inspired oxygen (PaO2: Fi2) ratio of 300 or less; and (2) a score of 4 or higher on the World Health Organization’s Ordinal Scale for Clinical Improvement, or a respiratory rate of 30 breaths per minute.^22^ An ARDS diagnosis was made according to the Berlin Definition criteria.^23^

### Interventions

All patients received standard of care as available and clinically indicated at the time of hospital admission without specific thresholds for method of supplemental oxygen (SpO_2_) delivery, method of ventilatory support, or medications. Available supplemental oxygen and ventilation interventions included nasal cannula (NC), simple face mask (FM), high flow nasal cannula (HFNC), non-invasive positive pressure ventilation (NIPPV), and intubation. Available COVID-19-directed medical therapies included vasopressors, sedative medications, hydroxychloroquine, glucocorticoids, tocilizumab, remdesivir, antibiotic therapy (including empiric treatment of community acquired pneumonia or hospital acquired pneumonia), and therapeutic anticoagulation.

### Data Collection

All categories of data collection were determined by a consensus group of internal medicine housestaff and intensivist faculty based on a review of the available literature at the time of the study. Data was entered into a secure database with specific parameters as defined by the authors.

Demographic data points were gathered in the following categories for all patients: age, sex, height, weight, body mass index (BMI), and pre-existing conditions. Lab value data points were gathered in the following categories for all patients on hospital days 0, 7, and 28: ferritin and D-dimer. Medications received by hospital days 0, 7, and 28 were recorded. Disease severity index scores were calculated at days 0, 7, and 28: sequential organ failure assessment (SOFA, with PaO_2_ from arterial blood gas, ABG, or if unavailable, calculated from pulse oximeter saturation [SpO_2, %_]), Acute Physiology and Chronic Health Evaluation II Score (APACHE II), and Ordinal Scale.^22,24^ Respiratory data points were gathered in the following categories for all patients at hospital days 0 and 14: mode of supplemental oxygenation/ventilation, fraction of inspired oxygen (FiO2, %), pH/PCO_2_ from blood gas (arterial if available, if not calculated from venous sample using the correction of +0.04 for pH and -0.06 for PvCO2), PaO_2_ (from ABG or if unavailable, calculated from the SpO_2_ at time of sampling), and P: F ratio.^22,24^ If patients were receiving IMV at hospital days 0 and/or 14 the following respiratory data points were also collected: respiratory rate (RR, breaths per minute), tidal volume (V_T_, mL), minute ventilation (V_E_, L/min), peak inspiratory pressure (PIP, cmH_2_O), mean airway pressure (MAP, cmH_2_O). These calculations were taken as an average of the highest and lowest values for that particular day. All additional demographic and intervention data not directly pertaining to this study are presented in the appendix.

Each patient’s medical record was reviewed for evidence of barotrauma, defined as radiographic evidence of air outside the pleura (noted as pneumothorax, pneumomediastinum, subcutaneous emphysema, or pneumopericardium) identified by a radiologist on chest x-ray (CXR), computerized tomography of the thorax (CTT), or computerized tomography of the abdomen/pelvis (CTAP). It should be noted that radiographic studies of the abdomen/pelvis were pursued for non-respiratory indications such as bowel obstruction or abnormalities with liver function tests, and extra-pleural air was noted regardless of being considered an incidental finding. No barotrauma events were found on post-procedural imaging for central line placement or intubation. For all patients who had sustained barotrauma, respiratory data as described above were also collected on the day of initial detection of barotrauma.

### Statistical Analysis

Patients were divided into 2 groups: those who had sustained barotrauma during admission and those who had not. The baseline characteristics of these 2 groups were compared, including age, sex, BMI, race/ethnicity, resuscitation status, pre-existing medical comorbidities, and whether or not they arrived from a skilled nursing facility (SNF). Continuous variables such as age and BMI were compared using a 2-sample t-test. Categorical variables such as sex, ethnicity, and presence of pre-existing comorbidities were compared using Fisher’s exact test.

Similarly, laboratory biomarkers and therapeutics received by these 2 groups were compared on hospital days 0, 7, and 28. Normally distributed continuous variables such as ferritin and D-dimer were compared using 2-sample t-test, and non-normally distributed continuous variables such as SOFA score and Ordinal Scale were compared using the Wilcoxon Rank-Sum test. Categorical variables such as type of therapy received were compared using Fisher’s exact test.

For patients who received IMV, the ventilator settings and respiratory data points were compared on hospital days 0 and 14 . Continuous variables such as PIP, MAP, V_E_ and V_T_ were compared using 2-sample t-tests. The distribution of ventilator modes for each group was compared using Fisher’s exact test. These ventilatory settings and respiratory data points were also compared for the day barotrauma was detected with day 14 of those who did not sustain barotrauma. Day 14 was chosen as the comparison time point because this was the median day of barotrauma observed at the time of this study’s design.

Overall length of stay, ICU length of stay, duration of intubation, mortality, and need for tracheostomy were calculated and compared between the barotrauma and non-barotrauma groups using Wilcoxon Rank-Sum due to the non-normal distribution.

Logistic regression was performed with barotrauma and mortality as outcomes, and intubation status as the covariates in the statistical model. We accept a type I error rate of 0.05 when determining the threshold for concluding significance when it does not exist. Stata (version 15, Stata, College Station, Texas) was used for all data analysis.

## Results

### Patient Characteristics and Outcomes

**Figure 1.**
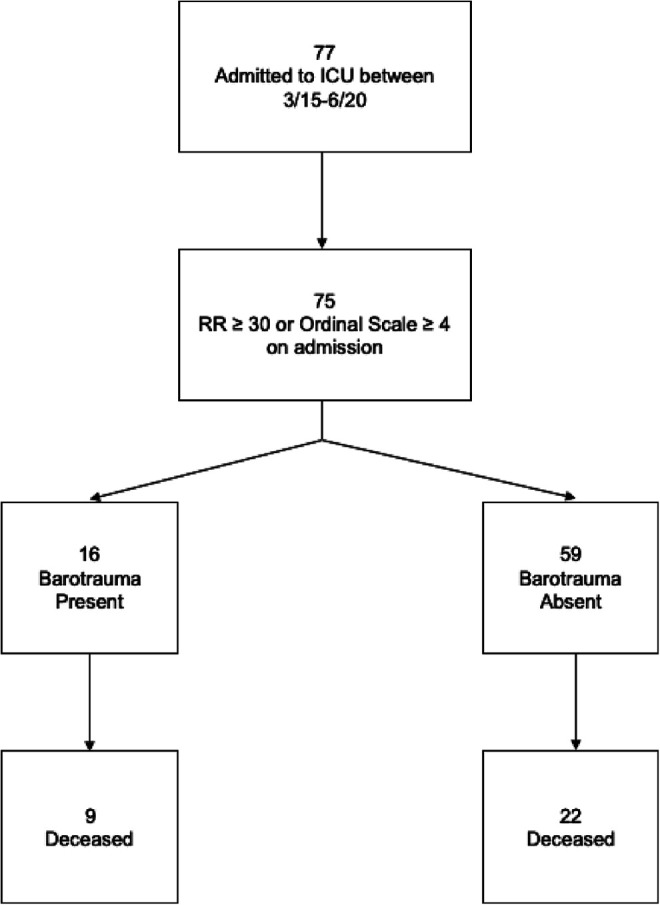
Mortality in patients with and without barotrauma.

**Figure 2.**
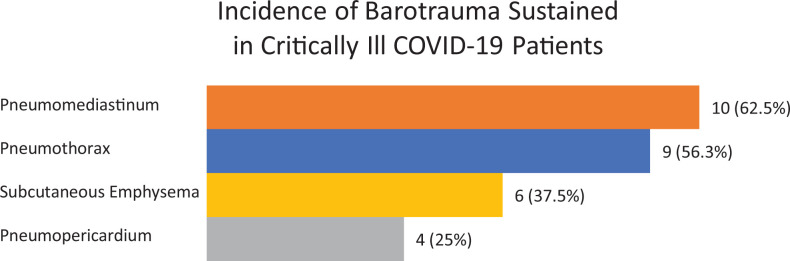
Incidence of barotrauma sustained in critically ill patients with COVID-19.

Between March 15 and June 20 of 2020, 77 patients were admitted to the ICU with COVID-19, 75 of whom met inclusion criteria. The 2 patients who did not meet inclusion criteria had a PaO2: Fi2 greater than 300 or Ordinal Scale score less than 4 and a respiratory rate in the less than 30 breaths per minute. Inclusion criteria and outcomes are depicted in Figure 1. There was a total of 16 patients who sustained one or more types of barotrauma: 9 with pneumothorax, 10 with pneumomediastinum, 6 with subcutaneous emphysema, and 4 with pneumopericardium (Figure 2). Of the 39 (52%) patients who received IMV, 13 (33%) sustained barotrauma. Of the 36 (48%) patients who received NIV, 3 (8%) sustained barotrauma. In patients receiving NIV who sustained barotrauma, 1 was receiving bilevel positive airway pressure (BIPAP) and 2 were receiving high-flow nasal canula (HFNC). Of note, none of these patients sustained barotrauma due to an immediate procedural complication based on imaging performed immediately after central line placement or endotracheal intubation.

### Between Group Differences

Between the 16 patients with barotrauma and 59 patients without barotrauma, there were no statistically significant differences in age, sex, BMI, race/ethnicity, and pre-existing conditions (Table 1). There were no significant differences in inflammatory markers (ferritin, d-dimer), ARDS severity (P: F ratio), intubation status, SOFA or Ordinal Scale score on hospital days 0, 7, and 28 between the barotrauma and non-barotrauma groups. On hospital day 0, there was a trend toward association between receiving hydroxychloroquine and barotrauma (38% in barotrauma group vs. 15% in non-barotrauma group, p = 0.08). By hospital day 28, there were a greater proportion of patients in the barotrauma group compared to the non-barotrauma group who had received convalescent plasma (56% vs. 27%, p = 0.04) and vasopressor therapy (63% vs. 32%, p = 0.04). There were no significant differences between the barotrauma and non-barotrauma group in receiving the following therapies: glucocorticoids, remdesivir, antibiotics, or tocilizumab on days 0, 7, or 28 (Table 2). Overall, there was a higher proportion of patients in the barotrauma group who had received therapeutic anticoagulation at any point during the hospitalization (81% vs. 47%, p = 0.02).

**Table 1.** Comparing Baseline Characteristics of Patients With and Without Barotrauma.

Characteristic		No barotrauma (N = 59)	Barotrauma (N = 16)	p-value
Age		60 (16)	54 (15)	0.19
Female Sex		31% (18)	13% (2)	0.21
BMI		29.3 (7.0)	29.2 (5.1)	0.93
Race/ethnicity	Hispanic	74% (44)	81% (13)	0.46
	Black	2% (1)	0% (0)	
	White	10% (6)	0% (0)	
	Asian	7% (4)	0% (0)	
	Unknown	7% (4)	19% (3)	
Code status	Full Code	80% (47)	63% (10)	0.19
	DNR/DNI	20% (12)	38% (6)	
**History of:**				
CAD		3% (2)	0% (0)	1.00
Hypertension		51% (30)	38% (6)	0.41
Type 2 Diabetes		58% (34)	56% (9)	1.00
COPD		2% (1)	0% (0)	1.00
Asthma		3% (2)	0% (0)	1.00
CKD		20% (12)	13% (2)	0.72
CVA		5% (3)	6% (1)	1.00
Cirrhosis		5% (3)	0% (0)	1.00
Psychiatric illness		10% (6)	0% (0)	0.33
Lung Disease		8% (5)	0% (0)	0.58
Tobacco use		20% (12)	13% (2)	0.72
Cancer		5% (3)	6% (1)	1.00
From SNF?		10% (6)	0% (0)	0.18

**Table 2.** Comparing Laboratory Markers and Therapeutics Received of Patients With and Without Barotrauma on Admission, Day 7, and Day 28.

Laboratory markers and therapeutics	No barotrauma N = 59	Barotrauma N = 16	p-value
***On Admission:***			
*Ferritin*	2200 (5600)	1500 (1200)	0.62
*D-dimer*	3.4 (5.1)	3.8 (7.0)	0.82
*Intubated*	10% (6)	13% (2)	0.68
*SOFA*	3 (2-4)	3.5 (2-4)	0.58
*Ordinal Scale*	4 (4-5)	4 (4-5)	0.47
*PaO_2_: FiO_2_ Ratio*	205 (98)	167 (90)	0.19
*Received Glucocorticoids?*	25% (15)	31% (5)	0.75
*Received Remdesivir?*	3% (2)	6% (1)	0.52
*Received Vasopressors?*	12% (7)	0% (0)	0.33
*Received Antibiotics?*	93% (55)	100% (16)	0.57
*Received Therapeutic Anticoagulation?*	12% (7)	19% (3)	0.44
*Received Hydroxychloroquine?*	15% (9)	38% (6)	0.08
*Received Convalescent Plasma?*	5% (3)	0% (0)	1.00
*Received Tocilizumab?*	0% (0)	0% (0)	
***Day 7:***			
*Ferritin*	2200 (6200)	1300 (1100)	0.55
*D-dimer*	4.7 (5.5)	6.6 (6.7)	0.27
*Intubated*	36% (21)	67% (10)	0.04
*SOFA*	3 (2-4)	4 (2-4)	0.86
*Ordinal Scale*	5 (4-6)	6 (5-6)	0.12
*Received Glucocorticoids?*	63% (37)	69% (11)	0.77
*Received Remdesivir?*	10% (6)	13% (2)	0.68
*Received Vasopressors?*	27% (16)	44% (7)	0.23
*Received Antibiotics?*	88% (52)	94% (15)	1.00
*Received Therapeutic Anticoagulation?*	39% (23)	50% (8)	0.57
*Received Hydroxychloroquine?*	46% (27)	56% (9)	0.58
*Received Convalescent Plasma?*	37% (22)	50% (8)	0.40
*Received Tocilizumab?*	7% (4)	25% (4)	0.06
***Day 28:***			
*Ferritin*	480 (110)	1200 (900)	0.22
*D-dimer*	5.3 (6.7)	6.8 (7.0)	0.78
*Intubated*	36% (21)	56% (9)	0.16
*SOFA*	2 (2-11)	8 (5-10)	0.51
*Ordinal Scale*	2 (1-8)	6.5 (3.5-7.5)	0.16
*Received Glucocorticoids?*	47% (28)	56% (9)	0.58
*Received Remdesivir?*	12% (7)	13% (2)	1.00
*Received Vasopressors?*	32% (19)	63% (10)	0.04
*Received Antibiotics?*	69% (41)	81% (13)	0.53
*Received Therapeutic Anticoagulation?*	39% (23)	69% (11)	0.05
*Received Hydroxychloroquine?*	41% (24)	44% (7)	1.00
*Received Convalescent Plasma?*	27% (16)	56% (9)	0.04
*Received Tocilizumab?*	12% (7)	25% (4)	0.23
***At any point during hospitalization:***			
*Received Glucocorticoids?*	73% (43)	75% (12)	1.00
*Received Remdesivir?*	14% (8)	13% (2)	1.00
*Received Pressors?*	44% (26)	63% (10)	0.26
*Received Antibiotics?*	100% (59)	100% (16)	1.00
*Received Anticoagulation?*	47% (28)	81% (13)	0.02
*Received Hydroxychloroquine?*	47% (28)	56% (9)	0.58
*Received Convalescent Plasma?*	62% (24)	75% (9)	0.50
*Received Tocilizumab?*	12% (7)	25% (4)	0.23

### Ventilator Mode and Support

There was no difference between peak inspiratory pressure (PIP), mean airway pressure (MAP), peak end expiratory pressure (PEEP), ARDS severity evidenced by P: F ratio (PaO_2_: FiO_2_), minute ventilation (V_E_), and tidal volume (V_T_) that patients in each group received on hospital days 0 and 14 (Table 3). The mean time from admission to the day of barotrauma detection was 13.6 days, median 11 days (interquartile range 5-16 days). Ventilation characteristics were compared between the barotrauma group on the day barotrauma was detected and the non-barotrauma group on day 14 (Table 3). The distribution of ventilator modes between the barotrauma and non-barotrauma group were statistically different (p = 0.049): in the barotrauma group, there was a higher proportion of patients in volume control (VC, 38% vs. 25%) and airway pressure release ventilation (APRV, 38% vs. 0%), whereas in the non-barotrauma group, there was a higher proportion of patients in pressure control mode (PC, 75% vs. 23%). In the barotrauma group the mean PIP was 29.4 cm H_2_O, mean MAP was 19.4 cm H_2_O, mean PEEP was 12 cm H_2_O, mean P: F ratio was 116, and mean TV was 6.8mL/kg ideal body weight. For the 3 cases of barotrauma sustained with NIV, we were unable to directly measure the respiratory data in regard to airway pressure.

**Table 3.** Comparison of Ventilator Characteristics With and Without Barotrauma on Day 0, Day 14, and Day of Barotrauma.

Ventilator characteristics		No barotrauma N = 59	Barotrauma N = 16	p-value
***Day 0:***				
*Ventilator mode*	VC	67% (4)	0% (0)	0.07
	PC	17% (1)	0% (0)	
	APRV	0% (0)	100% (2)	
	T-piece	17% (1)	0% (0)	
*PaO_2_: FiO_2_ Ratio*		205 (98)	167 (90)	0.19
*Peak Inspiratory Pressure*		25.0 (1.4)	25.3 (8.8)	0.95
*Mean Airway Pressure*		10.4 (0.8)	19.3 (14.5)	0.23
*Positive End Expiratory Pressure*		7.0 (2.4)	6.5 (2.1)	0.82
*Minute Ventilation (L/min)*		14.1 (2.5)	9.8 (2.5)	0.12
*cc/kg (Ideal Body Weight)*		8.4 (2.5)	7.1 (2.1)	0.55
***Day 14:***				
*Ventilator mode*	VC	22% (2)	25% (2)	1.00
	PC	67% (6)	75% (6)	
	CPAP	11% (1)	0% (0)	
*PaO_2_: FiO_2_ Ratio*		179 (103)	109 (47)	0.03
*Peak Inspiratory Pressure*		35.7 (9.3)	32.6 (9.2)	0.38
*Mean Airway Pressure*		20.1 (6.3)	19.7 (5.4)	0.78
*Positive End Expiratory Pressure*		11.8 (5.5)	14.1 (2.9)	0.29
*Minute Ventilation (L/min)*		13.7 (4.9)	11.0 (1.2)	0.11
*cc/kg (Ideal Body Weight)*		7.7 (2.6)	6.4 (1.2)	0.16
***Comparison on day of Barotrauma***		***Day 14***	***Day of Barotrauma***	
*Ventilator Mode*	VC	25% (2)	38% (5)	0.049
	PC	75% (6)	23% (3)	
	APRV	0% (0)	38% (5)	
*Peak Inspiratory Pressure*		35.7 (9.3)	29.4 (7.5)	0.08
*Mean Airway Pressure*		20.1 (6.3)	19.4 (6.2)	0.77
*Positive End Expiratory Pressure*		11.8 (5.5)	12 (5.5)	0.94
*PaO_2_: FiO_2_ Ratio*		146 (92)	116 (74)	0.37
*Minute Ventilation (L/min)*		13.7 (4.9)	12.2 (4.3)	0.45
*Tidal volume (mL/kg ideal body weight)*		7.7 (2.6)	6.8 (1.5)	0.31

### Outcomes

Barotrauma was associated with an increased length of hospital stay (26 days vs 14 days, p < 0.001), increased ICU length of stay (17 days vs 7 days, p = 0.003), and increased proportion of receiving a tracheostomy (19% vs. 2%, p = 0.03). There was a trend toward a higher median number of days intubated (12.5 days vs 7 days, p = 0.13). There was a trend toward a higher mortality in the barotrauma group compared to the non-barotrauma group (56% vs. 37%, p = 0.25). These findings are summarized in Table 4. Intubation was found to have an associated odds ratio (OR) of 5.5 (1.4-21, 95% CI) with barotrauma, and an OR of 8.9 (3.0-27, 95% CI) with mortality. Barotrauma, however, did not predict mortality (p = 0.17).

**Table 4.** Key Outcomes With and Without Barotrauma.

Outcome		No barotrauma N = 59	Barotrauma N = 16	p-value
*Length of Stay*		14 (9-19)	26 (23-45)	<0.001
*ICU Length of Stay*		7 (3-13)	17 (15-30.5)	0.003
*Days Intubated*		7 (6-11)	12.5 (10-20)	0.13
*Received Tracheostomy*		2% (1)	19% (3)	0.03
*Disposition*	SNF	10% (6)	0% (0)	0.18
	Home	44% (26)	25% (4)	
	OSH	9% (5)	19% (3)	
	Deceased	37% (22)	56% (9)	
*Mortality*		37% (22)	56% (9)	0.25
*28-Day Mortality*		31% (18)	25% (4)	0.77

## Discussion

In this study we observed a high incidence of barotrauma in patients with COVID-19 who were receiving IMV as well as NIV. In our cohort, we observed barotrauma in 33% of patients receiving IMV and 8% of patients receiving NIPPV, a total of 21% of all patients who were admitted to our ICU and met inclusion criteria. These findings demonstrate a much higher incidence of barotrauma than described in the original ARDSnet protocol (5-8%).^6^

Barotrauma has a known predictive value in length of hospital stay, morbidity, and mortality for patients experiencing ARDS.^5^ Similarly, in our cohort this was no different, we found there to be a significant increase in ICU length of stay and hospital length of stay, as well as a trend toward an increased duration of requiring ventilatory support. In our study, we found that mechanical intubation was significantly associated with sustaining barotrauma as well as with mortality. While we did not find a statistically significant correlation between barotrauma and mortality, these findings suggest that barotrauma portends a less favorable clinical outcome. A key limitation of our study is the relatively small sample size; as a single-center study, we are likely under-powered to detect the effects of barotrauma when compared to prior studies that have investigated barotrauma in SARS and MERS.^20,25^

The relationship between the ventilator and a critically ill patient is highly complex and individualized. Likewise, there are many factors, several unknown, that predispose patients with severe ARDS to developing barotrauma. After the initial ARDSNet protocol in 2000, it has been universally accepted that large tidal volumes, 12cc/kg ideal body weight, is associated with increased risk of barotrauma, whereas low tidal volume ventilation at 6cc/kg is viewed as more lung protective.^6–11,26^ Despite this widely practiced method of ventilatory support in ARDS, the rates of barotrauma in COVID-19, both in this study and others, such as the New York cohort (15% receiving IMV and 24% total separate events), are significantly higher.^16^ This was also the case with severe other viral respiratory illnesses such as SARS (34%), MERS (30%), and H7N9 (44%).^16,17,20,21^ In ARDS due to bacterial pneumonia and sepsis, the rates of barotrauma are much lower than these viral infections,^9,14,27^ which begs the question, is ARDS due to viral illness, or more specifically COVID-19 caused by some unique mechanism.^28^

It has been postulated, that COVID-19-induced ARDS (CARDS) is a unique disease state with a novel pathophysiology.^23,28,29^ One such proposal suggests CARDS consists of 2 distinct phenotypes, an “L” and an “H” type, where patients with “type L” ARDS have low elastance (high compliance), normal lung volume on imaging, and low response to PEEP due to absence of recruitable alveoli, as compared to “type H,” which is more “classic ARDS” characterized by high elastance (low compliance), low lung volume, and high PEEP responsiveness due to availability of recruitable alveoli.^29–31^ Supporters of this hypothesis advocate for low PEEP and high driving pressures in patients with the “type L” phenotype, which deviates from evidence-based practice of low tidal volume ventilation.^6–10^ It should be recognized with caution that this is a proposed mechanism and that ventilator management of a critically ill patient with severe hypoxemic respiratory failure requires individualized bedside assessment of airway pressures, pressure waveform, and ventilator synchrony as to avoid iatrogenic complications of mechanical ventilation.^32^ It is also important to note that ARDS is a heterogeneous disease with multiple pathologic stages starting with inflammation-induced leakage of proteinaceous material into the alveoli and resulting in severe fibrosis.^33^ This fibrosis ultimately causes poor compliance, which increases lung stress and strain, placing patients at an increased risk for barotrauma and volutrauma, regardless of inflammatory etiology.^34–36^

In our study, we interestingly found there to be no difference in MAP, PIP, PEEP, V_T_, or V_E_ at days 0 or 14 in patient who suffered barotrauma compared to those who did not, which argues that there may be other factors at play. There was a significant association between the use of APRV and the development of barotrauma, despite there being no overall difference in MAP. The reason for this is unclear. Perhaps the prolonged distending pressure and inspiratory time on the level of a damaged, leaky alveolus leads to further inflammation and rupture, or possibly that our measurement of MAP did not fully account for the spontaneous breaths in addition to our ventilatory settings, something that can be highly variable between patients.

One possible explanation for the association between barotrauma and COVID-19 is that this virus causes a heightened inflammatory response, more so than other infectious etiologies due to our lack of prior immunity, that manifests itself as rampant, diffuse alveolar damage. Since the pandemic began, several studies have looked at various cytokine levels in patients with COVID-19 and found that tumor necrosis factor alpha (TNFα) and interleukin-6 (IL-6) are routinely elevated.^37^ In extreme incidences, excessive cytokine production can result in macrophage activation syndrome (MAS) or hemophagocytic lymphohistiocytosis (HLH), conditions in which there is uncontrolled activation and proliferation of macrophages and lymphocytes due to impaired NK-cell cytotoxicity and regulation resulting a relative “immunoparalysis.”^37,38^ In addition to the viral pathology itself, there is also an association between IMV and increased cytokine production, namely TNFα and IL-6, that may be proportional to PEEP and tidal volumes.^39–41^ In animal models, TNFα has been thought to induce apoptosis perhaps predisposing these patients to alveolar rupture further implicating the role of this particular inflammatory process in severe COVID-19.^40–43^ This suggests that the alveolar stress from IMV may directly cause or worsen the inflammatory response seen in COVID-19 with worsening inflammation and alveolar damage.

Although purely speculative, the high incidence of barotrauma in COVID-19, particularly in the patients receiving NIV, may be the closest clinical link between the inflammatory pathway in COVID-19 and a unique physiology. Although, if barotrauma is a marker of poor pulmonary compliance, ARDS and the proposed CARDS share a final common pathway. Thus, the greatest clinical challenge with COVID-19 is not necessarily proving a novel phenotype for ARDS, rather it is trying to determine the phase of ARDS that a patient may be experiencing without having a precise understanding of the time course for the inflammatory process in each patient.

In our study we used ferritin and D-dimer as surrogate markers of inflammation and saw no difference in levels between the barotrauma and non-barotrauma cohorts, though these are values that have unclear significance during a severe respiratory infection. Given the limited resources at a county hospital during the height of the pandemic we were unfortunately unable to routinely check TNFα and IL-6 levels. One thing we did note was a correlation between having received therapeutic anticoagulation and risk of barotrauma. While some patients at our institution received this medication based on a d-dimer cutoff rather than objective evidence of venous thromboembolism, this may represent a specific subpopulation of patients with a heightened inflammatory response that was a predisposing factor for the barotrauma rather than the anticoagulation itself. Interestingly we did not notice a difference in disease severity by SOFA, APACHE, or Ordinal Scale which may also be surrogate markers of severe inflammation. Lastly, we did not notice any difference between specific COVID-directed therapies such as hydroxychloroquine, remdesivir, convalescent plasma, or glucocorticoids, which was not surprising as anyone sick enough to be admitted to the ICU routinely received these medications unless there was a contraindication.

### Limitations

As we are still discovering the ventilatory parameters and biomarkers that characterize this novel disease, there are possible confounders that this study is unable to assess, particularly in terms of the absence of predictive factors for barotrauma. The uniqueness of COVID-19 also makes finding an appropriate control cohort quite difficult. As more is learned about the optimal ventilation settings, disease pathophysiology, and treatment options for COVID-19, future studies should examine specific data points with more intentionality for the predictors of barotrauma, comorbidities, and mortality. Furthermore, the trends seen in this small single-center sample, particularly the risk of mortality from barotrauma and the possible association between hydroxychloroquine and barotrauma, may be significant in a larger cohort comprised of multiple centers. For the time being, however, data collection should continue to include typical metrics for ventilator settings, ARDS and disease severity indices, and the use of novel treatment modalities to learn more about the comorbidities and causes of severe disease.

## Conclusions

The pathophysiology of ARDS is extraordinarily complex and poorly understood. It remains to be seen if there is something inherently unique with COVID-19 that predisposes patients to heightened inflammation and worsened respiratory mechanics as compared to other causes of ARDS. But, regardless of the mechanism, the disproportionately high rate of barotrauma seen with COVID-19 must be considered as barotrauma correlates with longer ICU stay and hospitalization, and, perhaps most importantly a trend toward higher mortality. More work with laboratory and clinical studies is needed to understand this disease, its physiology, and its treatment. In the meantime, it is critical the medical community rely on evidence-based medicine, such as low lung volume ventilation, and individualized bedside care for the critically ill patient.
